# A needs assessment for a minor eye condition service within Leeds, Bradford and Airedale, UK

**DOI:** 10.1186/s12913-019-4448-8

**Published:** 2019-08-29

**Authors:** Alexander G. Swystun, Christopher J. Davey

**Affiliations:** School of Optometry and Vision Science, University of Bradford, Richmond Road, Bradford, BD7 1DP UK

**Keywords:** Needs assessment, MECS, Minor eye condition service, Optometry, Primary care, PEARS

## Abstract

**Background:**

There are a number of limitations to the present primary eye care system in the UK. Patients with minor eye conditions typically either have to present to their local hospital or GP, or face a charge when visiting eye care professionals (optometrists). Some areas of the UK have commissioned enhanced community services to alleviate this problem; however, many areas have not. The present study is a needs assessment of three areas (Leeds, Airedale and Bradford) without a Minor Eye Conditions Service (MECS), with the aim of determining whether such a service is clinically or economically viable.

**Method:**

A pro forma was developed for optometrists and practice staff to complete when a patient presented whose reason for attending was due to symptoms indicative of a problem that could not be optically corrected. This form captured the reason for visit, whether the patient was seen, the consultation funding, the outcome and where the patient would have presented to if the optometrists could not have seen them. Optometrists were invited to participate via Local Optical Committees. Results were submitted via a Google form or a Microsoft Excel document and were analysed in Microsoft Excel.

**Results:**

Seventy-five percent of patients were managed in optometric practice. Nine and 16% of patients required subsequent referral to their General Practitioner or hospital ophthalmology department, respectively. Should they not have been seen, 34% of patients would have presented to accident and emergency departments and 59% to their general practitioner. 53% of patients paid privately for the optometrist appointment, 28% of patients received a free examination either through use of General Ophthalmic Service sight tests (9%) or optometrist good will (19%) and 19% of patients did not receive a consultation and were redirected to other providers (e.g. pharmacy, accident and emergency or General Practitioner). 88% of patients were satisfied with the level of service. Cost-analyses revealed a theoretical cost saving of £3198 to the NHS across our sample for the study period, indicating cost effectiveness.

**Conclusions:**

This assessment demonstrates that a minor eye condition service in the local areas would be economically and clinically viable and well received by patients.

## Introduction

Minor Eye Conditions Services (MECS, also known as Primary Eye-care Acute Referral Schemes: PEARS) have been commissioned by Clinical Commissioning Groups (CCGs) in some areas of England, Wales and Northern Ireland. These enhanced optical services are commissioned at a local level to enable appropriate use of community resources to manage minor eye problems within routine optometric practice. Although detection and management of certain eye diseases is core competence for UK trained optometrists, accreditation is typically attained through online training [1]. Once this is completed, optometrists are able to offer NHS funded eye care beyond the scope of a general ophthalmic service (GOS) sight test in the form of a MECS appointment. Specifically, MECS aim to offer rapid access to professional eye care, thereby reducing unnecessary referrals into hospital ophthalmology departments. In turn, this is expected to reduce referral-related patient anxiety and will change the case-mix of overburdened ophthalmology departments to be more appropriate to secondary care [1–5].

Elderly patients make up the largest proportion of health service users [6], which combined with an increasingly ageing UK population is increasing the demand on hospital services. Ophthalmology departments are no exception [7, 8]. A number of reports have pointed to the conclusion that the overburdening of secondary eye care services directly results in a negative impact on patient safety and treatment [8–14]. Patient safety, combined with issues of accessibility, sustainability and convenience require the current practice of delivering eye care within a hospital setting to be reviewed [11–14].

One way of reducing the demand on hospital ophthalmology departments is to promote the management of certain eye conditions by optometrists [7]. In line with this, optometrists within ophthalmology departments are increasing in responsibility to facilitate the efficiency of doctors’ clinics [15]. As this increase in practitioner scope is limited to optometrists within a hospital setting, the demand on hospital ophthalmology departments and costs to the NHS remain unchanged. Although there are a limited number of studies evaluating MECS in different regions, it is typically reported that after a MECS consultation approximately 20% and 9% of patients require a hospital ophthalmology and general practitioner (GP) appointment, respectively [16–20]. It has been reported that this results in a reduction in GP and ophthalmology outpatient appointments [19, 21]. This reduction in unnecessary appointments results in a greater number of patients who require specialist care to receive it. Furthermore, two studies [22, 23] have shown that approximately 25% of patients attending specific ophthalmology accident & emergency (A&E) departments could have been successfully managed by an optometrist. These studies point to the conclusion that redirection of these patients to an optometrist could reduce the number of patients presenting to A&E with ocular issues.

According to ‘Annex A: The national prices and national tariff workbook of the National tariff payment system 2017/18 and 2018/19’,the current initial outpatient attendance fee for a single professional ophthalmology appointment is £139 with a follow up fee of £53. Similarly, a visit to A&E involving investigation starts from £93 [24]. In contrast, CCGs that have commissioned MECS in regions neighbouring the area of the present study receive remuneration of £40 (Wakefield), £44 (Harrogate) and £46 (Huddersfield). Similarly, the cost of a GP appointment is approximately £30 [25]. Given this lower average appointment cost within primary, relative to secondary, care successful management of eye issues within primary care could reduce the costs associated with managing this cohort of patients depending on how much demand increases due to currently unmet need.

Although the financial cost of a GP appointment is lower than the typical cost of a MECS assessment, GPs typically do not possess the necessary equipment and/or skills for investigation and intervention of ophthalmic problems. Specifically, it has been reported that GPs, on average, received 8 days of ophthalmology training at undergraduate level [26, 27]. Beyond this, 96% of GPs received no further ophthalmological training [28]. The end result is that, in one survey, 78% of general practitioners felt that their training on ophthalmology was inadequate [29].

NHS ‘red flags’ exist for GPs when examining patients with ophthalmological symptoms. Specifically, if a patient presents with any of the following acute signs or symptoms: sudden appearance of flashes or floaters, abnormal pupil reactions, moderate to severe pain or photophobia, marked redness of one eye, reduced visual acuity, reduced visual field, haloes around lights or foreign bodies, an urgent referral into the ophthalmology department is recommended [28, 30, 31]. It is expected that after examination by an optometrist, a lower number of patients would be referred, relative to when based solely upon presenting symptoms.

Currently, there are no Minor Eye Condition Services in Bradford, Airedale or Leeds. As such, two local optical committees (LOCs) commissioned this audit to investigate the need for a MECS scheme in the locality.

## Method

Bradford LOC and Leeds LOC both contacted their databases of optometrists with details of the needs assessment and invited local performers of optometric services to participate. The duration of the Bradford study was 6 weeks: running from 29/5/18 to 9/7/18. Leeds LOC conducted their study from 15/02/18 to 31/03/18 for a period of 6 weeks and 3 days.

The inclusion criteria was defined as any patient attending the participating optometric practice whose reason for visiting was due to symptoms indicative of a problem that cannot be corrected by spectacles/contact lenses. This definition is based on advice from the Association of Optometrists. Specifically, “Patients presenting with clear ocular medical concerns requesting a sight test for reasons (such as sticky red eye, foreign bodies and requests for a procedure, for example, if a patient’s doctor has advised a visual field check for driving) should be told that a GOS sight test is inappropriate and that they should be either treated privately in your practice or directed to hospital eye services or their GP as appropriate” [32]. The present study includes patients who are redirected before seeing the optometrist (e.g. by reception).

Results from Leeds were submitted through a Microsoft Excel document with drop down boxes and results from Bradford were submitted anonymously from a computer or mobile device via a Google form although an Excel spreadsheet was offered. The same questions and response options were used in both regions and there was free text box for any additional information the optometrist deemed appropriate.

## Results

**Fig. 1:**
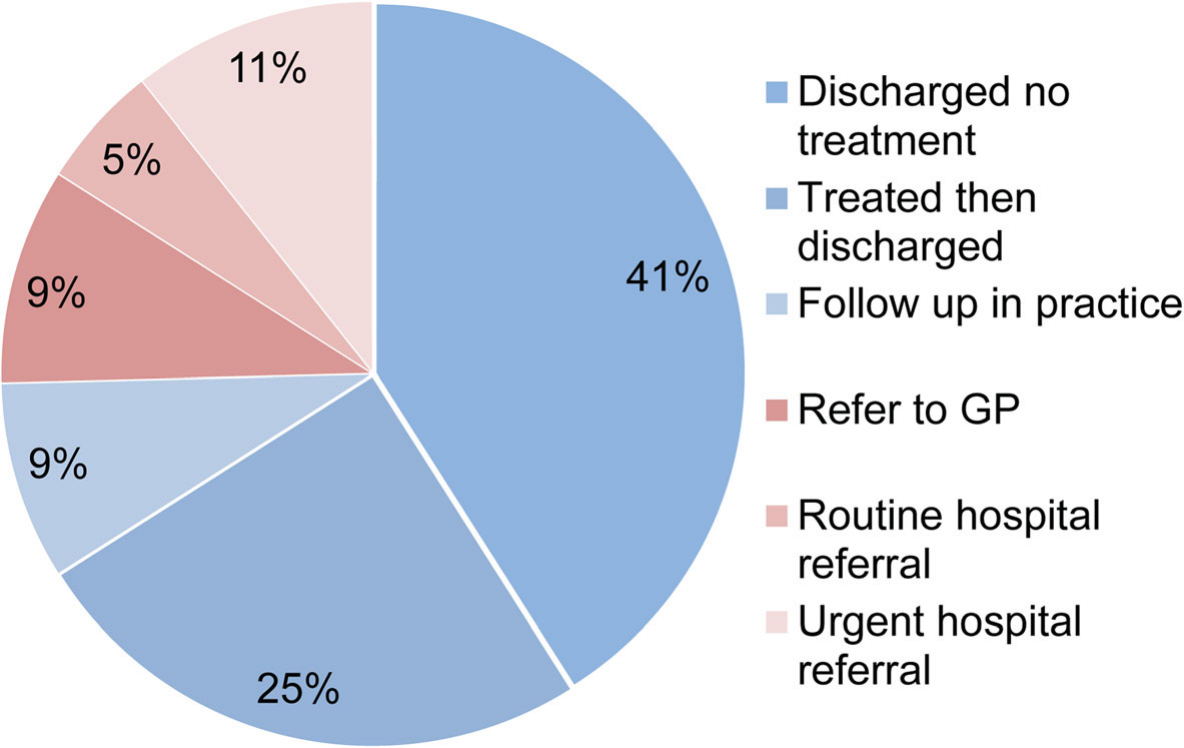
Appointment outcome (*n* = 244)

Responses from 105 patient encounters from 12 optometry practices within Leeds and 184 patient encounters from 34 optometrists within Bradford were received through the duration of the study. Following the examination, 75% of patients seen did not require intervention beyond the level of a primary care optometrist. 16% of patients were subsequently referred into hospital ophthalmology department and 9% required an onward referral to the GP (Fig. 1).

**Fig. 2:**
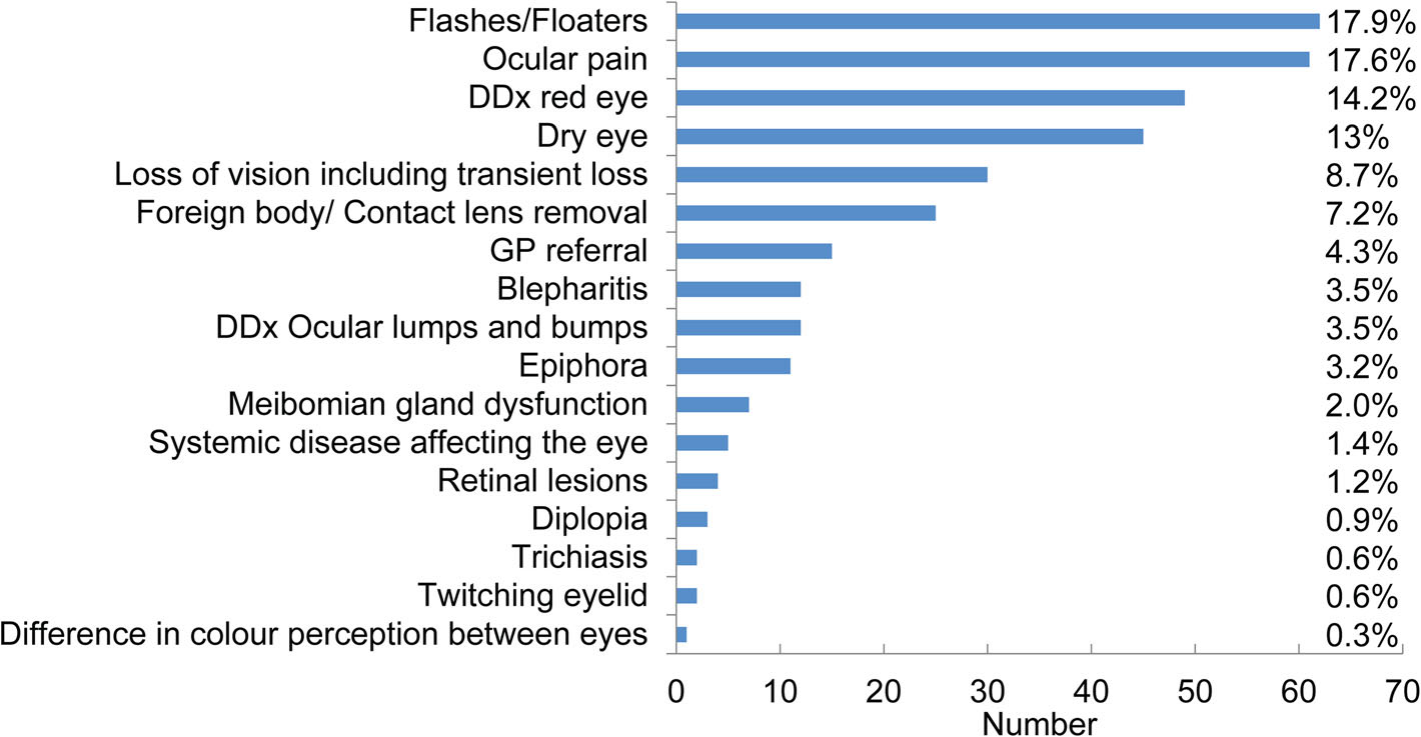
Reasons for obtaining an appointment (DDx = Differential diagnosis of, *n* = 346)

Optometric practice dealt with a range of acute eye problems. Figure 2 details the presenting complaints(s).

Patients typically presented to their optometrist with symptoms of an anterior eye problem (48%), potential posterior eye issues were relatively less common (19%). The remainder of presentations (33%) were ambiguous as to their location prior to seeing the optometrist (e.g. GP referral).

**Fig. 3:**
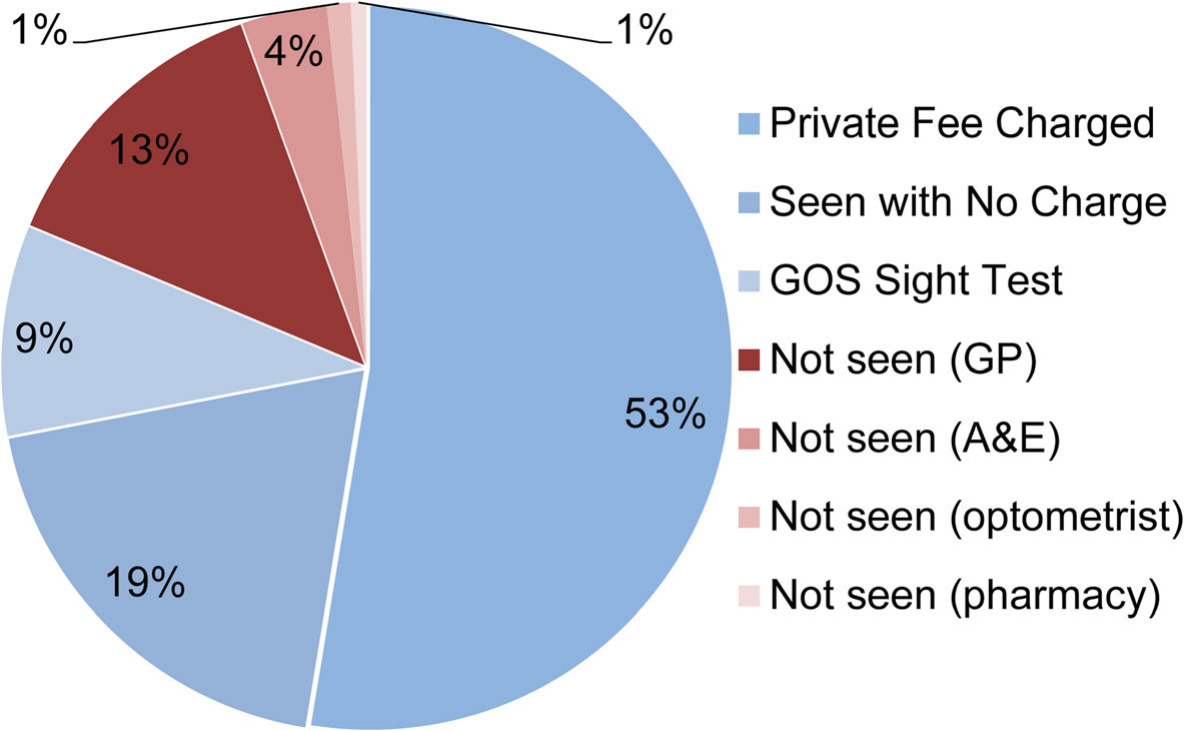
How was the consultation was funded (*n* = 289)

In the majority of cases, the patient paid a fee to access the optometrists’ service (53%). A number were seen at no charge, either as optometrist good-will (19%) or using a GOS claim (9%). The remainder of patients declined an appointment (19%) (Fig. 3).

Only six patient encounters specifically recorded the reason for the patient not receiving a consultation. Although only a small number, this was typically due to unwillingness to pay (4/6).

**Fig. 4:**
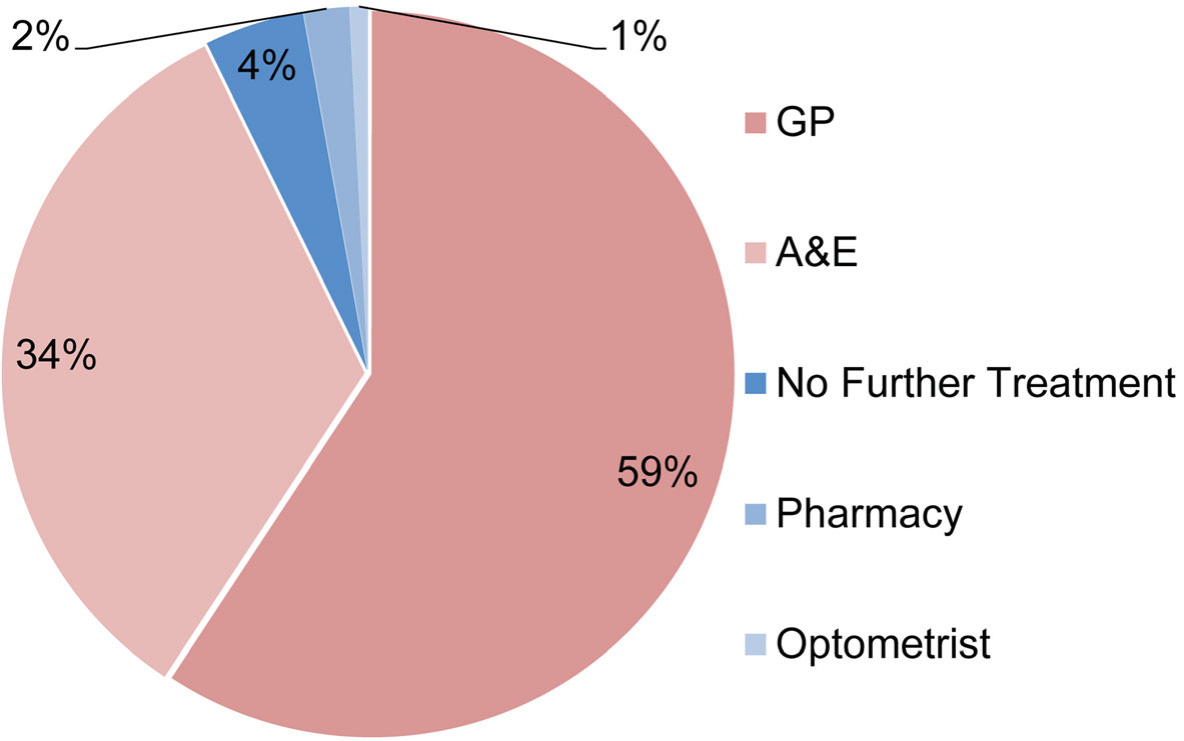
Alternative source of treatment if the optometrist was unable to see the patient (*n* = 248)

Perhaps unsurprisingly, the majority of patients presenting with an acute eye problem would have sought alternative treatment if they were unable to obtain an appointment with the optometrist (96%). Unmet demand is categorised as patients who have accessed a specific service that would not have accessed any alternative service. In the present study this could be classed as the proportion of patients that accessed an appointment with an optometrist that would not have otherwise sought professional advice and/or treatment (4%) (Fig. 4).

Sixty-two precent of patients presenting in the present study either had GP red flags [28, 30, 31] or were referred to an optometrist by the GP for a second opinion. If there was no community optometric service for seeing these patients, this large cohort of patients would have required the GP to refer into a hospital ophthalmology department; in many cases this would have been unnecessary. The results of this are particularly apparent when examining patients presenting with symptoms of flashing lights and/or floaters. Of the 55 patients presenting with flashing lights and floaters that were provided with a private optometric consultation, a GP would have been recommended to refer 100% urgently into ophthalmology [28, 30]. In the present study, after optometric examination it was found that 78% of these patients did not require ophthalmological intervention and were subsequently discharged with advice. Only 22% had signs of an underlying pathology requiring referral into hospital ophthalmology departments. Eighty-one patients presented to the optometrist with red flag symptoms excluding flashing lights and/or floaters (total red flag patients = 135). Nine further patients were referred to the optometrist by the GP for unspecified red flags or a second opinion. The presenting symptoms and outcome are detailed in Table 1.

**Table 1:** How patients presenting with ‘red flags’ were managed

Presenting Reason	Number Seen	Number Referred to Ophthalmology
Ocular Pain	30	3
Combinations ^a^	17	3
Foreign Body/ CL removal	14	1
Vision Loss	14	8
GP Referral	9	0
Marked Red Eye	6	2
Total	90	17

^a^’Combinations’ refers to more than one presenting reason. For example, a patient presenting with both a marked red eye and vision loss

Of the 55 red flag patients in the present study who would have sought the advice of the GP as an alternative to the optometrist, the optometrists referred 9 (16%) for an ophthalmological opinion. This details that a number of patients would have been referred to secondary care unnecessarily.

**Fig. 5:**
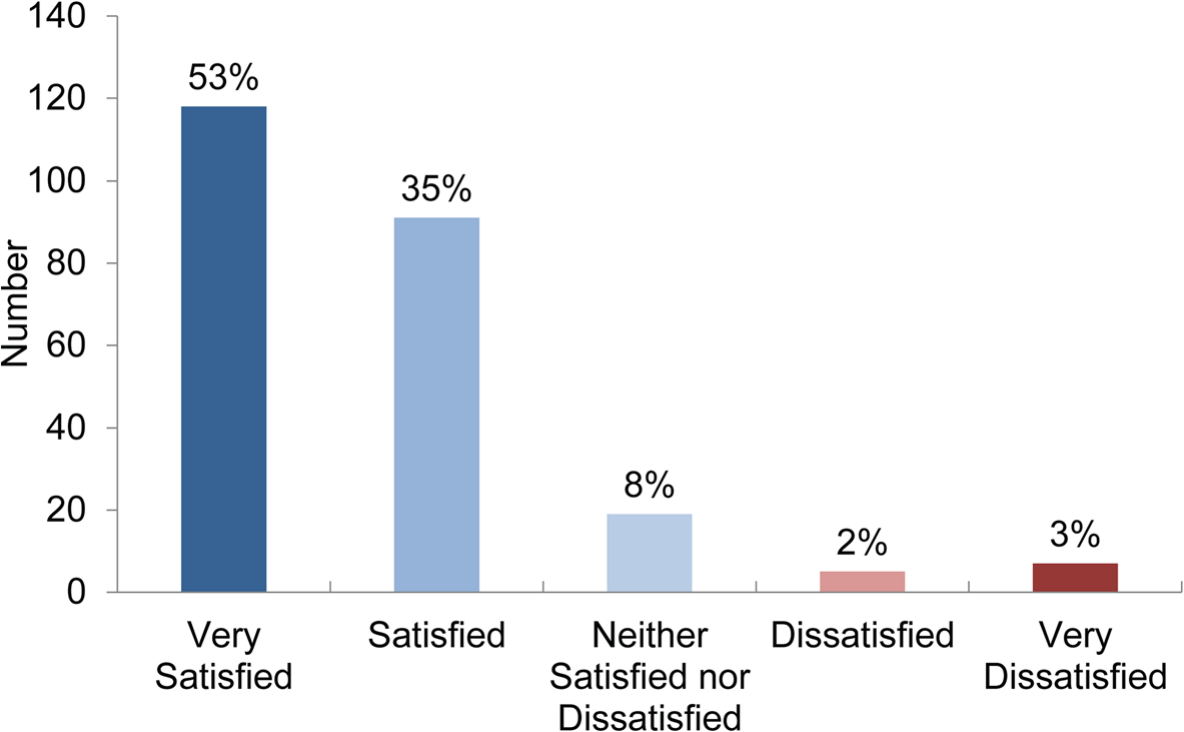
Satisfaction level with the service provided. (*n* = 239)

Patient satisfaction with the private MECS services was generally high (88%). From the patients who experienced dissatisfaction with the service (5%, *n* = 12), this most commonly occurred in instances where the patient had not received an appointment and were redirected (*n* = 7). The remainder of patients were indifferent about the level of service provided (8%) (Fig. 5).

### Cost analysis

For this cost analysis, of the 289 patient responses that were recorded, those that did not receive an appointment with the optometrist are excluded (*n* = 54). Furthermore, those who were seen, but had incomplete data (i.e. alternative source of treatment not filled in) were also excluded (*n* = 20). This leaves 215 patients of which, 133 presented with symptoms that are considered red flags. For this cohort of patients it is recommended that they will be referred into a hospital ophthalmology department [28, 30, 31]. For the present calculation, we will assume that 100% of the patients with red flags that visited the GP received this referral (*n* = 55). Although A&E doctors are likely to follow the same protocol as GPs for red flag symptoms, the present cost analysis adopts a conservative approach by assuming that the A&E doctor seeing these patients was an eye specialist and successfully managed these patients at first visit (*n* = 76). Similarly, we will assume that when a red flag patient would have seen another optometrist (if the first optometrist had been unable to see them), their condition did not require a referral (*n* = 2). Appointment costs are based on those mentioned earlier and a visit to the pharmacy is assumed to cost £0 and a figure of £46 is assumed for optometric reimbursement to reflect the highest cost of the scheme in neighbouring regions (Table 2).

**Table 2:** Cost analysis of the present study

*N* = 215	With Scheme	Without Scheme
Not Seen/ Pharmacy	0 x £0^i^ = £0.00	14 x £0 = £0.00
GP	15 x £30^ii^ = £450.00	123 x £30 = £3690.00
A&E	0 x £93^iii^ = £0.00	76 x £93 = £7068
Ophthalmology Referral	35 x £139^iv^ = £4865.00	55 x £139 = £7645.00
Optometrist	215 x £46^v^ = £9890.00	2 x £0 = £0.00
Total	£15,205.00	£18,403.00

^i^The cost to the NHS of seeing a community pharmacist, or if the patient doesn’t see anyone, is assumed to be £0. ^ii^A GP visit is costed at £30 [25]. ^iii^An A&E visit costs £93 [24]. ^iv^The cost of a first ophthalmology appointment is £139 [24]. ^v^The cost of a MECS appointment is presumed to be £46 which represents the highest first visit cost in neighbouring areas

The results of the present study show that in the 6 week timescale, with a relatively small number of participating optometrists there was a theoretical cost saving to the NHS of £13,088 as optometrists were seeing the patients and not redirecting them to GPs or A&E. In this example, a MECS scheme costing £46 per episode would have resulted in a theoretical cost saving of £3198 to the NHS. In this instance, optometric remuneration of less than £60.87 per MECS appointment would have resulted in a cost-saving. In reality, due to over demand for GP and secondary care resources, a MECS scheme may not reduce costs to the CCG, but does, however, result in a more appropriate case mix in secondary care that is cost-effective. Reduced costs may also be achieved by patients seeing an optometrist, nurse or other health care professional in place of an ophthalmologist within the hospital ophthalmology departments. However, with the present secondary care funding structure this could still be classed as an ‘ophthalmology led’ service and may not result in any decrease in costs to the CCGs.

## Discussion

At the time of writing, Bradford, Leeds and Airedale do not have a MECS commissioned. The results from the present study indicate that a MECS scheme would receive high patient satisfaction, while concurrently reducing the number of unnecessary presentations of eye conditions to general practice and secondary care. Furthermore, community management of minor eye conditions appears cost effective. Although important, cost is not the sole factor in determining viability of local enhanced services. Patient safety, satisfaction and service efficiency must be considered.

Getting it right first time is proposed to reduce waiting times, provide cost savings and improve the patient journey [33–35]. The present study supports this statement by demonstrating that a number of patients who would have seen the GP and subsequently been referred to ophthalmology did not require any treatment (84%). This result is perhaps unsurprising given the small amount of ophthalmological training UK GPs receive [26–29]. In line with this, a recent study on stakeholder attitudes towards MECS in Lewisham and Lambeth report that GPs support MECS, stating that ‘MECS would improve care and the patient journey’ [1].

Reports of the absolute cost savings of MECS are inconclusive. Whilst the PEARS in Wales incurs an increase in costs of approximately £12 per episode [19], reviews of the MECS in Lambeth and Lewisham found that cost savings were 0.6 and 16.9% respectively, relative to a control region in close proximity (Southwark) that didn’t have a MECS service [21]. Specifically, costs increased in the control region by 3.1%, whereas in Lambeth costs increased 2.5%. Lewisham, on the other hand, had cost savings of 13.8%. Whereas the data from the PEARS covers the whole of Wales, the data from England is limited to two areas with differing service specifications [21]. The differing results between these two areas highlight the dangers in generalising across the whole of England due to varying demographics. Importantly, however, it has been reported that MECS have been found to be cost-effective, irrespective of absolute cost savings [19, 21, 36].

In the present study, after receiving a privately funded appointment, 25% of patients required a further appointment from a healthcare professional (GP or ophthalmologist). In contrast, prior to the MECS appointment 93% of patients would have presented to the GP or A&E. Whilst there are very few studies on the unmet need of ophthalmology services [37], the amount of patients that would not have sought an alternative form of treatment in the present study was low (4%). These findings support the premise that MECS would reduce unnecessary referrals into hospital ophthalmology departments. Specifically, for patients presenting with symptoms of flashing lights and/or floaters, 78% of these were retained in primary care optometry: 22% were referred onto hospital ophthalmology. This figure in line with a number of studies citing the prevalence of retinal tear/breaks/detachment or other conditions requiring ophthalmological opinion is present in 13–27.1% of patients presenting with flashing lights and/or floaters [38–42]. As unnecessary (false-positive) referrals to hospital departments have been reported to cause negative psychological consequences to the patients [4, 5, 43], reducing the number of false-positive referrals into secondary care is expected to reduce the amount of referral-associated anxiety. An advantage of providing enhanced eye care within the community allows patients to have care closer-to-home with a more flexible appointment booking system. Beyond the financial sustainability, as in the UK there are significantly greater numbers of optometrists [44], relative to ophthalmologists [45], it is also expected that care by optometrists in the community would be more sustainable for the workforce.

Although a small number of patients presented with loss of vision (*n* = 14), they were typically referred to either ophthalmology (*n* = 8) or to their GP (n = 1). Only 5 of these patients could be successfully managed in optometric practice. This indicates that there could be certain conditions that should bypass the optometrist and be directed directly to secondary care. Further work, however, is needed to explore this.

The present study supports previous findings demonstrating that cost is a factor influencing whether a patient will present to an optometrist [46, 47]. Although only a small number of presentations explicitly recorded the reason for the patient declining an appointment, the majority (4/6) recorded that the patient declined due to the fee. In these instances the patient was redirected to free-to-access health care (A&E, GP, and Pharmacy). Although this number is too small to draw any conclusions, it is in line with the core principles and values of the NHS: Specifically, that health care will be provided free at the point of delivery and not based on the ability to pay [48]. Previous studies have indicated that optometrist participation in these schemes partially depends on financial remuneration [1]. For the reasons aforementioned, to ensure widespread optometrist participation and public engagement it is important that any MECS is appropriately funded. The cost analysis of the present study reveals that community eye care could be an effective use of the finite resources of the NHS.

### Limitations

A limitation of the present study is that only a sample of optometrists in the area participated which increases the likelihood of a self-selection bias being present in these data. This, however, could be mirrored by the self-selection of those optometrists who decide to participate in enhanced community services like MECS. Attempts were made to quantify how many optometrists in total were practising in the area however these data were not available from either Local Optical Committees or NHS Primary Care Support England. The geographical location, optometrist experience or practice type may also result in bias in the clinical decision making [49, 50].

In the present study, as participating optometrists knew that their results would be closely audited, this may have influenced their clinical decision making resulting in an observer effect. The impact of this may be considerable given that participating optometrist also knew that these results could influence whether or not a MECS would be commissioned in these areas. Regular and continuing audit, therefore, is essential to assess long term effectiveness and efficiency of any enhanced scheme.

A further limitation of the present study is that it the false-negative outcome of the patients managed by primary care optometrists was not measured. Although the results of the present study were broadly similar between Leeds, Bradford and Airedale, further work is needed to assess the impact that a MECS would have in other areas of the UK, due to varying local referral guidelines and demographics.

## Conclusion

The present study supports the view that improvements in primary eye care could be made by using optometry based enhanced services for the management of acute eye problems. It would be expected that this service would alter the case mix of referrals into hospital ophthalmology departments making it more appropriate to secondary care. Furthermore, this study provides support for the notion that a MECS in Bradford, Leeds and Airedale would contribute towards efficient use of finite NHS resources while retaining high levels of patient satisfaction.

## Data Availability

The datasets used and/or analysed during the current study are available from the corresponding author on reasonable request.

## Abbreviations

A&E: Accident and Emergency
CCG: Clinical Commissioning Group
DDx: Differential Diagnosis
GOS: General Ophthalmic Service
GP: General Practitioner
LOC: Local Optical Committee
MECS: Minor Eye Condition Service
NHS: National Health Service
PEARS: Primary Eyecare Acute Referral; Scheme
